# Hydrogen Gas Presents a Promising Therapeutic Strategy for Sepsis

**DOI:** 10.1155/2014/807635

**Published:** 2014-04-16

**Authors:** Keliang Xie, Lingling Liu, Yonghao Yu, Guolin Wang

**Affiliations:** ^1^Department of Anesthesiology, General Hospital of Tianjin Medical University, Tianjin 300052, China; ^2^Tianjin Institute of Anesthesiology, Tianjin 300052, China

## Abstract

Sepsis is characterized by a severe inflammatory response to infection. It remains a major cause of morbidity and mortality in critically ill patients despite developments in monitoring devices, diagnostic tools, and new therapeutic options. Recently, some studies have found that molecular hydrogen is a new therapeutic gas. Our studies have found that hydrogen gas can improve the survival and organ damage in mice and rats with cecal ligation and puncture, zymosan, and lipopolysaccharide-induced sepsis. The mechanisms are associated with the regulation of oxidative stress, inflammatory response, and apoptosis, which might be through NF-**κ**B and Nrf2/HO-1 signaling pathway. In this paper, we summarized the progress of hydrogen treatment in sepsis.

## 1. Introduction

Sepsis is defined as the presence (probable or documented) of infection together with systemic manifestations of infection [1]. Severe sepsis is defined as sepsis plus sepsis-induced organ dysfunction or tissue hypoperfusion [1]. Sepsis-induced tissue hypoperfusion is defined as infection-induced hypotension, elevated lactate, or oliguria [1]. Septic shock is defined as sepsis-induced hypotension persisting despite adequate fluid resuscitation [1]. Sepsis and its various adverse sequelae, such as septic shock, acute respiratory distress syndrome (ARDS), and multiple organ dysfunction syndrome (MODS), continue to be a leading cause of mortality in intensive care unit (ICU) and a major public health burden throughout the world [2]. With recent dramatic advances in powerful antibiotics and monitoring devices, the mortality rate has been decreased over the past half century [3]. However, the number of people dying from sepsis continues to rise annually owing to the increasing morbidity. It is estimated that about 18 million cases of severe sepsis occur annually worldwide and the incidence rate of sepsis is increased by 1.5% ~ 8% annually [4, 5]. At present, there are more than 1,000,000 cases of severe sepsis among hospitalized patients each year in the USA with a total annual cost of $16.7 billion [6, 7]. In China, the occurrence rate of severe sepsis in surgical ICU is 8.68% with a hospital mortality rate of 48.7% [8, 9].

The pathogenesis and mechanisms of sepsis are complex and not fully understood, which include the excessive release of inflammatory cytokines, the action of oxidative stress (excessive release of reactive oxygen species, ROS), intestinal bacteria and endotoxin translocation, neutrophil dysfunction, microcirculatory impairment, mitochondrial dysfunction, the imbalance between oxygen supply and oxygen consumption, immune and metabolic disorders, and coagulation disorders [10–15]. Our previous studies have found that the uncontrolled inflammatory response and oxidative stress are crucial to the pathogenesis of sepsis, MODS, and ultimately death [16–18]. In addition, it should be noted that genetic variations partially determine individual susceptibility to sepsis. An increasing number of candidate genes have been implicated in sepsis susceptibility, such as macrophage migration inhibitory factor, plasminogen activator inhibitor 1, protein C, and miRNA [19].

However, even when sepsis is timely recognized, there is no effective therapy to sepsis, except antibiotics, fluids, and vasopressors [9, 20]. Disappointedly, undeniable successes in numerous animal studies are not consistent with that in clinical trials, such as direct anti-inflammatory strategies including anti-TNF-*α*, IL-1-based therapies, high-dose corticosteroids, and administration of activated protein C, which make the researches about pathogenesis and clinical treatment of sepsis troubled [10, 21–23]. The novel interventional strategies of sepsis should be invented to reduce mortality in sepsis.

Molecular hydrogen (H_2_), the smallest, lightest, and most element in the universe, is colorless, odorless, and certain antioxidant. Many years ago, H_2_ was regarded as physiological inert gas without more attention from scientists because of relatively low solubility and the difficulty to be absorbed. In 1975, Dole et al. found that exposure to a mixture of 2.5 percent oxygen and 97.5 percent hydrogen at a total pressure of 8 atmospheres for periods up to 2 weeks would cause a regression of squamous cell carcinoma via antioxidant effect [24]. In 1997, Shirahata et al. [25] reported that electrolyzed-reduced water, which dissolved large amounts of H_2_, had the ability to protect DNA from oxidative damage, suggesting that it could reduce the risk of life style-related diseases and cancer. H_2_ has also been used in medical applications to prevent decompression sickness in deep-sea divers for safety profiles [26]. In 2001, Gharib et al. [27] reported that treatment with 0.7 MPa hydrogen in a hyperbaric chamber for 2 weeks had significantly protective effects towards schistosomiasis-associated chronic liver inflammation, which was associated with antioxidant and anti-inflammatory properties of H_2_. It is also proved that molecular hydrogen would directly react with the hydroxyl radical, a highly cytotoxic reactive oxygen species (ROS). In 2007, Ohsawa et al. [28] found that H_2_ could exert a therapeutic antioxidant activity by selectively reducing hydroxyl radical and peroxynitrite (another cytotoxic ROS) in vitro, making researches about molecular hydrogen become hot around the world. In recent years, many researchers have found that molecular hydrogen can attenuate multiple organ damage, such as brain, spinal cord, heart, lung, liver, kidney, pancreas, and intestine [28–34]. Besides, it is widely proved that H_2_ or H_2_-rich saline exerts an effective therapeutic role in many diseases including sepsis, ischemia-reperfusion injury, organ transplantation, stroke, MODS, type 2 diabetes, atherosclerosis, neurodegenerative diseases, and oxygen toxicity [28, 30, 31, 35–40]. However, the mechanisms by which molecular hydrogen provides beneficial effects on many disorders remain unclear, which would be associated with reduction of oxidative stress, inflammation, and apoptosis, as well as regulation of several important signaling pathways.

## 2. Advances in Hydrogen Treatment of Sepsis

We have made several studies about H_2_ treatment in animal models of sepsis. It is well known that cecal ligation and puncture (CLP) causes lethal peritonitis and sepsis due to a polymicrobial infection that is accompanied by multiple organ damage. We firstly investigate the possible therapeutic effects of H_2_ on sepsis in a murine model of moderate or severe CLP. For severe CLP (100% lethality), we ligate the distal three-quarters of the cecum and make a single puncture with a 20-gauge needle; for moderate CLP (30–40% survival), we ligate the distal one-half of the cecum and make a single puncture with a 21-gauge needle. We find that H_2_ inhalation starting at 1 and 6 h after CLP operation significantly improved the survival rate of septic mice with moderate or severe CLP in a concentration- and time-dependent manner [30]. Moreover, H_2_ inhalation at a therapeutic dose (2% and 4%) has no adverse effects on the saturation level of arterial oxygen and hemodynamic parameters. We further find that H_2_ treatment provides the beneficial effects on sepsis and sepsis-associated organ damage, including lung, liver, kidney, and brain [30]. Zymosan, a substance derived from the cell wall of the yeast* S. cerevisiae*, can lead to systemic inflammation by inducing a wide range of inflammatory mediators. The zymosan-induced generalized inflammation model has been widely used in many experimental studies for MODS. We also find that H_2_ improves survival rate and organ damage in zymosan-induced generalized inflammation model [16]. 2% H_2_ inhalation for 1 hour beginning at 1 and 6 hours after zymosan injection significantly improves the 14-day survival rate of zymosan-challenged mice from 10% to 70%. H_2_ treatment significantly mitigates the impairments of liver and kidney function in the zymosan-challenged mice [17]. Intratracheal administration of lipopolysaccharide (LPS), the major component of the outer membrane of Gram-negative bacteria, is a well-established model of acute lung injury. Using this model, we find 2% H_2_ or hydrogen-rich saline can exert protective effects in a mouse model of acute lung injury [29]. Meanwhile, combination therapy with H_2_ (2%) and hyperoxia (98%) increases the 14-day survival rate of moderate sepsis mice to 100% and the 7-day survival rate of severe sepsis mice from 0% to 70% and alleviates injuries of lung, liver, and kidney in moderate and severe sepsis [41]. Similarly, we find that 2% H_2_ inhalation significantly ameliorates short- and long-time cognitive function in sepsis survivors (unpublished data). In addition, we find that hydrogen-rich saline can significantly improve the outcome and cardiac function in a rat model of septic shock (unpublished data). Thus, H_2_ or hydrogen-rich saline may be an effective therapeutic strategy for patients with sepsis (Table 1).

## 3. Mechanisms about Hydrogen Treatment of Sepsis

### 3.1. Anti-Inflammatory Effects

Sepsis is associated with a systemic inflammatory response, mediated by vascular endothelial cells and innate immune cells, including neutrophils, macrophages, and monocytes [48]. The releases of proinflammatory cytokines and chemokines, including tumor necrosis factor- (TNF-) *α*, interleukin- (IL-) 1, IL-6, high-mobility group box- (HMGB-) 1, and monocyte chemoattractant protein- (MCP-) 1, as the most important cytokines mediating the acute phase of inflammatory response, normally triggers beneficial host innate immune response to confine the infection and tissue damage. However, in sepsis, the excessive and prolonged production of these cytokines can produce overwhelming inflammatory response, which is even more deadly than the original infection. These inflammatory cytokines can result in a variety of pathologic phenomena, including priming of the vascular endothelium by synthesis of adhesion molecules, activation of neutrophils, synthesis of cyclooxygenase products, generation of nitrous oxide, ROS, apoptosis, and induction of hypotension and shock-like state [49–52]. It is well known that the excessive production of proinflammatory cytokines causes capillary leakage, tissue injury, and lethal multiple organ failure in severe sepsis [10, 53, 54]. It is also reported that elevated proinflammatory cytokine levels directly correlate with severity and mortality in human sepsis [55, 56]. The proinflammatory cytokines also lead to activation of the complement and coagulation cascades [57]. Furthermore, proinflammatory cytokines can upregulate the expression of inflammatory mediators via positive feedback loop and, consequently, induce further detrimental phenomena [58].

HMGB1 is a member of the high-mobility group protein superfamily that has been widely studied as nuclear proteins that bind DNA, stabilize nucleosomes, and facilitate gene transcription [59]. HMGB-1 has been implicated in the pathogenesis of inflammatory diseases and proposed to be a crucial mediator in the pathogenesis of many diseases including sepsis, arthritis, cancer, autoimmunity diseases, and diabetes [60]. HMGB1 can interact with various receptors including RAGE, Toll-like receptor- (TLR-) 2, and TLR-4 to mediate chemotaxis and release of proinflammatory cytokines in monocytes/macrophages and delayed endotoxin lethality, which is required for the full expression of inflammation in animal models of endotoxemia, sepsis, and arthritis [59, 61]. Furthermore, targeting of HMGB1 with antibodies or specific antagonists has been found to have protective effects in established preclinical inflammatory disease models, including lethal endotoxemia and sepsis [62]. As the late inflammatory cytokine, HMGB1 plays a central role in the inflammatory response, becoming a key therapy to resolve inflammation [63, 64].

Our studies show that H_2_ inhalation can decrease the early proinflammatory cytokines (TNF-*α*, IL-1*β*, and IL-6) and late proinflammatory cytokine (HMGB1) in serum and tissues (lung, liver, and kidney) of preclinical animal models of sepsis [16, 29, 30, 41]. Furthermore, H_2_ treatment reduces the levels of chemokines (KC, MIP-1*α*, MIP-2, and MCP-1) in the bronchoalveolar lavage fluid of LPS-induced ALI mice. In addition, H_2_ treatment decreases LPS-induced neutrophils recruitment into the lungs [29]. All results demonstrate that H_2_ treatment downregulates the cytokines and chemokines in the different mouse models of sepsis. Therefore, it has been suggested to use molecular hydrogen as a new anti-inflammatory strategy.

Recently, some studies have shown that neuroinflammation in the central nervous system can cause brain damage in sepsis. The vast release of cytokines, such as TNF-*α*, IL-1*β*, and HMGB1, leads to alterations of cell function, the blood-brain barrier disruption, and brain dysfunction [65–67]. In particular, HMGB1, released from necrotic neurons via a NR2B-mediated mechanism, promotes cerebral edema via activation of microglial TLR4 and the subsequent expression of the astrocytic water channel, aquaporin-4 in traumatic brain injury [68]. We find that the blood-brain barrier disruption and cognitive dysfunction of sepsis animals are significantly alleviated by H_2_ treatment, which are associated with the decrease of the levels of proinflammatory cytokines (TNF-*α*, IL-1*β*, and HMGB1) in the cerebral tissue (unpublished data).

With the accumulation of knowledge regarding proinflammatory cytokines produced by the immune system, investigators focus on the physiological mechanisms that maintain homeostasis. So far, various endogenous anti-inflammatory mediators are proved to have a capacity to prevent proinflammatory cytokine-mediated diseases [48]. IL-10, as an important anti-inflammatory and immunosuppressant, can mediate the downregulation of inflammatory response by a variety of mechanisms. Importantly, IL-10 can inhibit the generation and release of a variety of cytokines in monocytes/macrophages, such as TNF-*α*, IL-1, IL-8, GM-CSF, and G-CSF [69]. IL-10 plays a protective role in the systemic inflammatory response. Combination therapy with H_2_ (2%) and hyperoxia (98%) significantly increases the IL-10 level in serum and tissues (lung, liver, and kidney) of septic mice with moderate or severe CLP [41].

### 3.2. Antioxidant Effects

Oxidative stress defines disequilibrium between the levels of produced ROS and the ability of a biological system to detoxify the reactive intermediates [70]. ROS can be generated through several pathways such as direct interactions between redox-active metals and oxygen species via reactions including the Fenton and Haber-Weiss reactions, or by indirect pathways involving the activation of enzymes such as nitric oxide synthase (NOS) or NADPH oxidases. Intracellular accumulation of ROS, such as superoxide anion, hydrogen peroxide, singlet oxygen, hydroxyl radical, and peroxy radical, can arise from toxic insults or normal metabolic processes. These species may perturb the cell's natural antioxidant defense systems, resulting in damage to all of the major classes of biological macromolecules, including nucleic acids, proteins, carbohydrates, and lipids [70]. Furthermore, hydroxyl radical is one of the strongest oxidant species and reacts indiscriminately with nucleic acids, lipids, and proteins [71]. One type of ROS can be converted into another type via antioxidant enzymes in vivo. For example, superoxide dismutase (SOD) converts superoxide anion radical into H_2_O_2_, which is detoxified into H_2_O by either glutathione peroxidase or catalase (CAT) [72]. Besides ROS, reactive nitrogen species (RNS) can mediate nitrosative stress. RNS are generated by the quick reaction of superoxide with nitric oxide (NO), which results in the production of large amount of peroxynitrite [73, 74].

In excess, ROS and their by-products that are capable of causing oxidative damage may be detrimental to tissues and organs [75]. Recently, some studies demonstrated that H_2_ would exert a therapeutic antioxidant activity by selectively reducing hydroxyl radicals (the most cytotoxic ROS) and effectively reverse tissue damage such as transient cerebral ischemia, neonatal cerebral hypoxia-ischemia, liver injury, lung injury, and myocardial injury induced by ischemia and reperfusion [31, 32, 76–78]. A growing number of studies have found that excessive production of ROS and RNS plays important roles in the pathogenesis of sepsis [79–81]. Therefore, scavenging ROS, RNS, and their by-products is a critical antioxidant process, which may be a good and critical measure for treating sepsis. We report that H_2_ treatment significantly decreases the levels of 8-iso-prostaglandin-F2*α* (8-iso-PGF2*α*) in serum, lung, liver, and kidney tissue, which could exactly reflect the level of oxidative stress [16, 29, 30]. The levels of 8-iso-PGF2*α* in serum and tissues are also reduced by combination therapy with H_2_ and O_2_ [41]. In addition, our researches find that H_2_ treatment can significantly improve the activities of antioxidant enzymes (SOD and CAT) in serum and organ tissues of mice of moderate and severe sepsis models [16, 30, 41]. These outcomes suggest that H_2_ treatment provides beneficial effects on sepsis and sepsis-associated organ damage, which are associated with downregulation of oxidative stress.

### 3.3. Antiapoptosis Effects

Apoptosis, the regulated destruction of a cell, is a complicated process [82]. Many pathways can lead to activation of cell death. Death proteases are homologous to each other and are part of a large protein family known as the caspases, and blocking caspases can rescue condemned cells from their apoptotic fate. Besides the caspases, mitochondria sequester is a potent cocktail of proapoptotic proteins. Most prominent among these is cytochrome C, the humble electron carrier. Cytochrome C is one of the components required for activation of caspase-9 in the cytosol. Bcl-2 family is intimately involved in the regulation of cytochrome C crossing the mitochondria.

In addition, a role for oxidative stress in apoptosis has been shaped by several independent observations. For many years, direct treatment of cells with oxidants like hydrogen peroxide or redox-active quinones was thought to exclusively cause necrosis, but more recent studies have shown that lower doses of these agents can trigger apoptosis [83].

Apoptosis is a common pathological basis of many diseases, which plays an important role in the development of various diseases. Xiang et al. [84] detect that 2% H_2_ inhalation markedly attenuates morphological liver injury and apoptosis by reducing lipid peroxidation such as MDA. Cai et al. [77] find that 2% H_2_ therapy in a duration-dependent manner significantly reduces the number of positive TUNEL cells and suppresses caspase-3 and caspase-12 activities in neonatal hypoxia-ischemia rat model. We find that H_2_ inhalation markedly inhibits pulmonary cell apoptosis by TUNEL staining in LPS-challenged mice. Similarly, the caspase-3 activity is significantly increased in the lungs of LPS-challenged animals, which is prevented by H_2_ treatment [29]. Moreover, 1.3% H_2_ can reduce the number of apoptotic positive cells and infarct sizes due to opening of mitochondrial *K*_ATP_ channels followed by inhibition of mPTP in the acute myocardial infarction and reperfusion model [85]. Besides, H_2_-rich saline may effectively decrease the degree of necrosis, apoptosis, and cell autophagy in rats with acute CO poisoning, which could be related to decrease in the content of Fe and increase in the content of serum Cu associated with free radical metabolism [86].

### 3.4. Signaling Pathways

NF-*κ*B transcription factors also regulate the expression of hundreds of genes that are involved in regulating cell growth, differentiation, development, inflammation, and apoptosis [87]. In quiescent cells, NF-*κ*B activity is principally regulated by the I*κ*B proteins, which possess ankyrin repeats and are generally inhibitory to DNA binding. The activity of the typical I*κ*Bs is controlled through phosphorylation by upstream I*κ*B kinases (IKKs). The canonical NF-*κ*B pathway is activated mostly by the stimulation of proinflammatory receptors, such as the TNF receptor superfamily, the Toll-like receptor family (TLRs), and cytokine receptors for the interleukins [88]. It is also activated by genotoxic agents as well. Phosphorylation of I*κ*B*α* on serines 32 and 36 by the IKK complex (primarily IKK*β*) targets it for ubiquitination. Subsequently the ubiquitinated I*κ*B*α* is degraded by the proteosome and this unmasks the DNA-binding activity of the p50/RelA heterodimer and also allows it to translocate to the nucleus where it can bind to *κ*B sites and activate gene transcription. It is well known that NF-*κ*B regulates gene expression of cytokines, chemokines, and adhesion molecules. Therefore, NF-*κ*B is increasingly recognized as a crucial player in many steps of regulation of inflammatory responses. Noncanonical NF-*κ*B activation is stimulated by specific TNF receptor family members that signal through the recruitment of TRAF2 and TRAF3 [88]. In addition, different target genes are differentially induced by distinct NF-*κ*B dimers. Furthermore, NF-*κ*B subunits also contain sites for phosphorylations and other posttranslational modifications which are important for activation and crosstalk with other signaling pathways [87]. Heme oxygenase-1 (HO-1) and apoptosis-associated factors, including TRAF-1 and Bcl-XL, are also mediated by NF-*κ*B [89]. A previous study indicates that H_2_ inhalation reduces epithelial apoptosis in ventilator-induced lung injury via NF-*κ*B activation [90]. H_2_ inhibits TNF-*α*-induced lectin-like oxidized LDL receptor-1 expression by inhibiting the phosphorylation of I*κ*B-*α* and activation of NF-*κ*B in endothelial cells [91]. Moreover, H_2_ can indirectly activate the NF-*κ*B signaling through reducing oxygen free radical [28]. However, in our study, H_2_ treatment inhibits the lung NF-*κ*B p65 nuclear translocation and DNA-binding activity in LPS-challenged mice [29].

Nuclear factor erythroid 2-related factor 2 (Nrf2) is an important cytoprotective transcription factor [92]. Nrf2 controls the coordinated expression of important antioxidant and detoxification genes (Phase II genes) through a promotor sequence termed the antioxidant response element (ARE). Phase II genes, including heme oxygenase-1 (HO-1), glutathione S-transferases (GSTs), and NAD(P)H quinine oxidoreductase, work in synergy to constitute a pleiotropic cellular defense that scavenges reactive oxygen/nitrogen species (ROS/RNS), detoxifies electrophiles and xenobiotics, and maintains intracellular reducing potential. HO-1 is an ubiquitous and redox-sensitive inducible stress protein that degrades heme to CO, iron, and biliverdin [93]. Some studies have found that Nrf2 is a novel regulator of the innate immune response that dramatically improves survival during experimental sepsis by protecting against dysregulated inflammation [94]. Heme oxygenase-1 (HO-1) and the product of its enzymatic reaction, CO, not only have beneficial anti-inflammatory properties, but also enhance bacterial clearance by increasing phagocytosis and the endogenous antimicrobial response [95]. Some researchers found that hydrogen treatment during exposure to hyperoxia significantly improved blood oxygenation, reduced inflammatory events, and induced HO-1 expression, which did not mitigate hyperoxic lung injury or induce HO-1 in Nrf2-deficient mice [96]. Previous studies investigate that hydrogen gas inhalation significantly promotes the expression of Nrf2 in septic organs including lung, liver, and kidney. However, hydrogen gas did not improve the survival rate in Nrf2-deficient mice [46, 97]. Moreover, H_2_ treatment dose-dependently attenuates the increased levels of proinflammatory cytokines and further increases the level of anti-inflammatory cytokine IL-10 with the increase of HO-1 protein expression and activity in LPS-stimulated RAW 264.7 macrophages [43]. Therefore, downstream molecules of Nrf2 signaling pathway play an important role in the pathophysiological process of sepsis.

## 4. Hydrogen-Rich Saline Exerts the Same Therapeutic Effect with H_2_

Hydrogen-rich saline, in which the concentration of hydrogen is more than 0.6 mmol/L, is easily and safely manufactured [29]. Hydrogen-rich saline can alleviate inflammatory response, inhibit cell apoptosis, and reverse oxidative stress to reduce organ injuries, which would be a good method for clinical application. Currently, it is generally accepted that hydrogen-rich saline exerts an effective therapeutic role in many disorders including sepsis, ischemia-reperfusion injury, allergy, and degenerative diseases [29, 98–100].

In our study, we find that hydrogen-rich saline has similar beneficial effects on LPS-induced lung injury as hydrogen inhalation, which are also associated with inhibition of infiltration of inflammatory cell and activation of NF-*κ*B [29]. In addition, hydrogen-rich saline effectively ameliorates hemodynamics, vascular reactivity in a dose-dependent manner in rat model of peritonitis-induced septic shock. Meanwhile, vital organ dysfunction, such as heart, lung, liver, and kidney, is significantly mitigated via resolving inflammatory responses and decreasing the iNOS expression [44]. Similarly, hydrogen-rich saline has potential protective effects against sepsis by decreasing proinflammatory responses, oxidative stress, and apoptosis in a rat model of polymicrobial sepsis [45]. In addition, hydrogen-rich saline markedly reverses cognitive impairment and mortality in a dose-dependent manner in rats submitted to sepsis by cecal ligation and puncture, which are relative to the suppression of oxidative stress and cell apoptosis [101].

## 5. Advantages of H_2_ or Hydrogen-Rich Saline Treatment of Sepsis

It is obvious that hydrogen is electronically neutral and has favorable distribution characteristics: it can penetrate biomembranes and diffuse into the cytosol, mitochondria, and nucleus [102]. Despite the moderate reduction activity of H_2_, its rapid gaseous diffusion might make it highly effective for reducing cytotoxic radicals. Besides, it stands to reason that H_2_ will react with only the strongest oxidants. H_2_ is mild enough not to disturb metabolic oxidation reduction reactions or to disrupt ROS involved in cell signaling—unlike some antioxidant supplements with strong reductive reactivity, which increase mortality, possibly by affecting essential defensive mechanisms. Thus, H_2_ treatment is advantageous for medical procedures without serious unwanted side effects [28]. Furthermore, H_2_ is neither inflammable nor explosive at low concentrations (<4.6% in air and 4.1% in pure oxygen) [28]. Moreover, only 2% hydrogen gas can have obvious protective effects on sepsis. Meanwhile, hydrogen-rich saline is also available and safe for medical applications.

## 6. Conclusion

Although recent treatment modalities and interventions have contributed to the improvement for sepsis patients, the high mortality rate of severe sepsis suggests the necessity for additional therapies. Recently, vigorous experimental studies have been undergone to identify effective therapy of molecular hydrogen for sepsis. What is more, this novel therapy may be tested in clinical situation in the future. However, we should deeply proceed to more experimental researches to investigate the plausible and comprehensive mechanisms of hydrogen to treat sepsis.

## Figures and Tables

**Table 1:** Effects of molecular hydrogen treatment on sepsis.

Drugs	Authors	Animal or cell type	Animal or cell model	Administration dose and way	Effect
2% hydrogen	Liu et al. (2013) [42]	Wister rats	15 mg/kg LPS (septic shock)	Inhalation for 2 h after LPS injection	Anti-inflammatory (IL-6, IL-8, TNF-*α*, and MPO) Antioxidant (*·*OH, MDA, and SOD) Inhibition of apoptosis (Fas, Bcl-2)

Hydrogen-rich saline	Chen et al. (2013) [43]	RAW 264.7 macrophages	1 mg/mL LPS	Incubation with hydrogen-rich saline	Anti-inflammatory (TNF-*α*, IL-1*β*, HMGB1, and IL-10) Nrf2/HO-1 signaling pathway

Hydrogen-rich saline	Xie (2013) [44]	Wister rats	CLP	5 mL/Kg or 10 mL/Kg	Anti-inflammatory (MPO) Antioxidant (iNOS)

2% H_2_ and 98% hyperoxia	Xie et al. (2012) [41]	C57BL/6 mice	CLP	2% H_2_ and 98% hyperoxia for 3 h and 6 h after CLP	Anti-inflammatory (TNF-*α*, HMGB1, IL-10, and MPO) Antioxidant (CAT, SOD, and 8-iso-PGF2*α*)

Hydrogen-rich saline	Li et al. (2013) [45]	Sprague-Dawley rats	CLP	5 mL/Kg hydrogen-rich saline at 0, 6, and 18 h after CLP	Anti-inflammatory (TNF-*α*, IL-6, HMGB1, IL-10, HMGB1, and MPO) Antioxidant (MDA, SOD)

2% hydrogen	Xie et al. (2012) [46]	C57BL/6 mice	CLP	2% H_2_ inhalation for 1 h and 6 h after CLP	Nrf2/HO-1 signaling pathway

2% hydrogen or hydrogen-rich saline	Xie et al. (2012) [29]	C57BL/6 mice	25 ug LPS (acute lung injury)	2% H_2_ inhalation for 1 h and 6 h after LPS given or 10 mL/Kg hydrogen-rich saline	Anti-inflammatory (TNF-*α*, IL-1*β*, IL-6, IL-10, KC, MIP-1*α*, MIP-2/MCP-1, and MPO) NF-*κ*B signaling pathway Inhibition of apoptosis (TUNEL, caspase-3)

2% hydrogen	Xie et al. (2010) [30, 47]	C57BL/6 mice	CLP	2% H_2_ inhalation for 1 h and 6 h after CLP	Anti-inflammatory (HMGB1, MPO) Antioxidant (CAT, SOD, and 8-iso-PGF2*α*)

2% hydrogen	Xie et al. (2010) [16]	ICR mice	1 g/Kg zymosan (inflammation model)	2% H_2_ inhalation for 1 h and 6 h after zymosan injection	Anti-inflammatory (TNF-*α*, HMGB1) Antioxidant (SOD, 8-iso-PGF2*α*)
